# GLT6D1 rs1537415 G Allele and Advanced Periodontitis: A Meta-Analysis

**DOI:** 10.7759/cureus.114871

**Published:** 2026-08-20

**Authors:** Theofanis A Karaoulas, Christoforos Xirouchakis, Chariklia Neophytou, Ioannis Fragkioudakis

**Affiliations:** 1 School of Dentistry, Aristotle University of Thessaloniki, Thessaloniki, GRC; 2 Preventive Dentistry, Periodontology and Implant Biology, Aristotle University of Thessaloniki, Thessaloniki, GRC; 3 Institute of Dentistry, Queen Mary University of London, London, GBR

**Keywords:** chronic periodontitis, genetic susceptibility, glt6d1, meta-analysis, periodontitis, rs1537415

## Abstract

This meta-analysis aimed to evaluate the association of the GLT6D1 rs1537415 G allele with susceptibility to periodontitis stage III/IV, grade B or C. Adhering to Preferred Reporting Items for Systematic reviews and Meta-Analyses (PRISMA) guidelines, three eligible studies were identified through comprehensive searches of PubMed, Web of Science, and Scopus. Data from 2,185 participants (668 cases and 1,517 controls) were analyzed using random-effects models to calculate odds ratios (ORs) and 95% confidence intervals (CIs). Heterogeneity was assessed using Chi² and I² statistics. Formal testing for publication bias was precluded by the small number of studies (n = 3). The meta-analysis demonstrated a significant association between the rs1537415 G allele and severe periodontitis, with a pooled OR of 1.58 (95% CI: 1.28-1.95). Subgroup analyses showed consistent associations in European and Sudanese populations, whereas no significant association was observed in the Brazilian cohort. Sensitivity analysis excluding the largest Genome-Wide Association Study (GWAS) attenuated the association to a non-significant level (OR = 1.37; 95% CI: 0.87 to 2.16), indicating that a single study largely drove the overall effect; the pooled estimate should therefore be interpreted with caution. The GLT6D1 rs1537415 G allele may represent a genetic risk factor for severe periodontal diseases, particularly stage III/IV and grade B/C, suggesting a possible role in immune modulation. Further research involving larger, multi-ethnic cohorts is crucial to validate these associations and explore gene-environment interactions.

## Introduction and background

Periodontitis is a chronic inflammatory disease that progressively destroys the supporting structures of teeth, including the periodontal ligament and alveolar bone. If untreated, periodontitis can lead to tooth loss and has been linked to systemic health complications such as diabetes and cardiovascular disease [1]. The disease arises from a complex interplay of genetic, microbial, and environmental factors, with genetic predisposition accounting for approximately 50% of individual susceptibility [2].

Genetic research in periodontitis has evolved considerably, moving from early linkage analysis [3] and candidate gene studies [4] to contemporary genome-wide association studies (GWAS) and next-generation sequencing (NGS) [5,6]. These approaches have enabled a much broader exploration of the genome, identifying thousands of genetic variants, particularly single-nucleotide polymorphisms (SNPs), potentially relevant to periodontitis. Bioinformatics resources such as the 1000 Genomes Project [7] and HapMap [8] have further improved the interpretation and cataloging of genetic variation across diverse populations. Applying these methods, a systematic review of GWAS on periodontitis analyzed 15 high-quality studies and identified 11 SNPs meeting genome-wide significance thresholds (P < 5 × 10⁻⁸), along with 41 SNPs of suggestive significance (P < 5 × 10⁻⁶) [9]. However, only three SNPs (rs4284742 [G], rs11084095 [A], and rs12461706 [T], all from the sialic acid-binding Ig-like lectin 5 (*SIGLEC5*) gene region) were consistently reported across studies, underscoring the heterogeneity of genetic findings in this field. Other genome-wide significant SNPs have also been linked to periodontitis: rs729876 on chromosome 16p13.12 shows trans-expression quantitative trait loci (trans-eQTL) effects on homeobox C10 (*HOXC10*) in monocytes, with possible effects on inflammation and fibrinolysis, while rs16870060 on chromosome 8q22.3 has tissue-specific effects related to inflammation and immune modulation [10]. Despite these findings, the functional implications of many of these SNPs remain inadequately understood.

Among the genetic loci associated with periodontitis, the glycosyltransferase 6 domain containing 1 (*GLT6D1*) gene has received particular attention. *GLT6D1* was first identified as a genome-wide significant susceptibility locus for periodontitis in 2010, in a GWAS of German and Dutch patients with aggressive periodontitis. It was subsequently examined in a Sudanese cohort with aggressive periodontitis and a Brazilian cohort with advanced periodontitis. More recently, a larger European meta-GWAS of severe periodontitis (Stage III/IV, Grade C, diagnosed at ≤35 years) confirmed *GLT6D1* among its significant genetic loci and additionally identified the Fc fragment of IgE receptor Ig (*FCER1G*) and hemicentin 2 (*HMCN2*), both linked to oral barrier stability and wound healing [6,11]. However, findings regarding the rs1537415 allele's effects have not been entirely consistent across populations; while some European studies suggest it to be protective, other populations have shown conflicting results [12,13].

*GLT6D1* encodes glycosyltransferase 6 domain-containing protein 1, an enzyme thought to be involved in glycosylation processes that support epithelial barrier integrity, stabilize cell-surface receptors, and help preserve extracellular matrix (ECM) architecture [14]. In periodontal tissues, proper glycosylation is proposed to support immune receptor signaling and cell-cell interactions that regulate leukocyte recruitment and limit excessive inflammatory damage [10]. The rs1537415 variant is located in the second intron of *GLT6D1*, within a predicted binding site for the GATA binding protein 3 (GATA-3) transcription factor. Functional analyses have suggested that the G allele reduces the binding affinity of GATA-3 [14], a transcription factor important for T-helper 2 (Th2) cell differentiation and the induction of anti-inflammatory cytokines such as Interleukin (IL)-4, IL-5, and IL-13. This reduction in GATA-3 binding capacity has been proposed to impair Th2-mediated immune modulation, potentially shifting the host response toward a pro-inflammatory T-helper 1/T-helper 17 (Th1/Th17) profile characterized by elevated IL-1 beta, tumor necrosis factor-alpha (TNF-α), and matrix metalloproteinases [10]. Such changes are thought to promote chronic inflammation, ECM degradation, and accelerated alveolar bone loss, hallmarks of severe periodontitis [14,15]. Clinically, carriers of the rs1537415 G allele have been reported to more often present with Stage III/IV, Grade C disease, with clinical attachment loss and radiographic bone destruction disproportionate to local plaque accumulation [14,16], suggesting that the functional consequences of the variant may extend beyond host-microbe interactions to include immune dysregulation and impaired tissue repair.

Although rs4284742, rs11084095, and rs12461706 in *SIGLEC5 *were the SNPs most consistently replicated in the broader systematic review by Gao et al. [9], rs1537415 in *GLT6D1* was selected for this meta-analysis because of its strong and reproducible association with aggressive and advanced periodontitis across multiple GWAS, its proposed functional relevance in immune regulation via GATA-3 binding affinity, and the absence of a dedicated meta-analysis evaluating its effects across diverse populations. Despite this evidence, most studies to date have focused on Stage III/IV, Grade C disease, and the role of *GLT6D1* in more common [3], slower-progressing periodontitis subtypes remains underexplored; population-specific variation and methodological inconsistencies across studies further limit the generalizability of existing findings [4,5,17]. Clarifying whether this association is robust and reproducible across populations is clinically relevant, since a validated genetic marker could eventually support risk stratification and earlier, targeted preventive care in individuals at elevated genetic risk, before irreversible periodontal tissue destruction occurs.

Accordingly, the objective of this meta-analysis was to evaluate the association between the *GLT6D1* rs1537415 G allele and susceptibility to severe periodontitis (Stage III/IV, Grade B or C) across diverse populations. This was framed as a focused review question: in individuals with periodontitis (Population), how does carriage of the rs1537415 G allele (Exposure), compared with non-carriage (Comparison), relate to the odds of severe or advanced disease (Outcome)?

This review was previously posted as a preprint on Research Square (DOI: 10.21203/rs.3.rs-6953323/v1) in July, 2025.

## Review

Methods

Search Strategy

This study followed the Preferred Reporting Items for Systematic Reviews and Meta-Analyses (PRISMA) guidelines to ensure transparency, reproducibility, and methodological rigor [18] (for the complete checklist, see Appendices). The primary objective was to evaluate the association between the *GLT6D1* G allele (rs1537415) and periodontitis through a meta-analysis.

A structured search was conducted across the following databases: PubMed, Web of Science, and Scopus. The search strategy included keywords such as "*GLT6D1*," "rs1537415," "G allele," "aggressive periodontitis," "chronic periodontitis," "stage III," "stage IV," "grade B," "grade C," and "periodontitis." The search was limited to studies published in English. Additionally, reference lists of eligible articles were screened to identify further relevant studies (Table 1).

**Table 1:** Detailed search strategy

Database	Search Terms and Keywords	Filters Applied	Search Date
PubMed	("GLT6D1" OR "rs1537415" OR "G allele") AND ("periodontitis" OR "aggressive periodontitis" OR "chronic periodontitis" OR "stage III" OR "stage IV" OR "grade B" OR "grade C")	Language: English	August 2026
Web of Science	("GLT6D1" OR "rs1537415" OR "G allele") AND ("periodontitis" OR "aggressive periodontitis" OR "chronic periodontitis" OR "stage III" OR "stage IV" OR "grade B" OR "grade C")	Language: English	August 2026
Scopus	TITLE-ABS-KEY ("GLT6D1" OR "rs1537415" OR "G allele") AND ("periodontitis" OR "aggressive periodontitis" OR "chronic periodontitis" OR "stage III" OR "stage IV" OR "grade B" OR "grade C")	Language: English	August 2026
Reference Lists	Manual screening of reference lists of eligible articles	None	August 2026

Eligibility Criteria

Studies were included if they met the following criteria: (i) Published in peer-reviewed journals and reporting data on the prevalence of *GLT6D1* G allele carriers in cases of periodontitis and healthy controls (e.g., NGS, GWAS, case-control, cohort, or cross-sectional studies), (ii) utilized validated diagnostic criteria for periodontitis, encompassing both aggressive and chronic forms (e.g., American Academy of Periodontology/European Federation of Periodontology classifications), and (iii) provided sufficient information to calculate odds ratios (ORs) and 95% confidence intervals (CIs). Exclusion criteria included: (i) Studies lacking genotype frequency data for rs1537415, (ii) reviews, case reports, or studies without original data, and (iii) studies involving animal models or in vitro experiments. The detailed inclusion and exclusion criteria applied in this study are also presented in Table 2.

**Table 2:** Inclusion and exclusion criteria NGS: next-generation sequencing; GWAS: genome-wide association studies; AAP: American Academy of Periodontology; EFP: European Federation of Periodontology

Inclusion criteria	Exclusion criteria
Peer-reviewed studies (e.g., NGS, GWAS, case-control, cohort, cross-sectional) reporting prevalence of GLT6D1 G allele carriers in periodontitis cases and healthy controls.	Lack of genotype frequency data for rs1537415.
Use of validated diagnostic criteria for aggressive and chronic periodontitis (e.g., AAP/EFP classifications).	Reviews, case reports, or studies without original data.
Sufficient data to calculate odds ratios (ORs) and 95% confidence intervals (CIs).	Animal models or in vitro experiments.

Study Selection and Data Extraction

Two reviewers (TAK and CX) independently screened all titles and abstracts retrieved from the database searches to assess eligibility according to the predefined inclusion and exclusion criteria. Prior to formal screening, a calibration exercise was performed on a random sample of 20 articles to ensure consistent interpretation and application of the criteria, achieving full agreement before proceeding. Full-text screening of potentially eligible articles was also performed independently by the same two reviewers. Disagreements at any stage were resolved through discussion, and if consensus could not be reached, a third reviewer (IF) served as an adjudicator. This process minimized selection bias and enhanced methodological rigor. 

Two reviewers (TAK and CX) extracted the following data: genotype and allele frequencies of rs1537415, statistical results, including ORs, CIs, and p-values, and methods of genetic analysis (e.g., polymerase chain reaction (PCR), GWAS).

Statistical Analysis

Data were analyzed using the Review Manager (RevMan) software. The Mantel-Haenszel method with a random-effects model was employed to calculate pooled ORs and 95% CIs. All estimates were derived under a dominant (carrier) model, comparing individuals carrying at least one rs1537415 G allele (GG or GA genotype) with non-carriers (AA genotype). ORs and 95% CIs were calculated from 2x2 tables of carrier status by disease status, applied uniformly across all included studies. Heterogeneity among studies was assessed using the Chi² test and the I² statistic: I² > 50% was considered indicative of substantial heterogeneity. Publication bias was planned to be assessed using funnel plot symmetry and Egger's regression test; however, because fewer than 10 studies were included, these methods are underpowered and unreliable. A funnel plot was generated for transparency, but results should be interpreted with caution.

Risk-of-Bias Assessment

The quality of the included studies was assessed using the Q-Genie tool for evaluating genetic studies [19,20]. This tool examines several aspects of study design and methodology, including: adequacy of the hypothesis and rationale, classification and measurement of outcomes, description and comparison of study groups, disclosure of potential sources of bias, and assessment of statistical methods and power analysis. Q-Genie assists in determining the overall quality and reliability of genetic studies, thereby enhancing the robustness of meta-analysis findings.

Results

Study Selection

**Figure 1:**
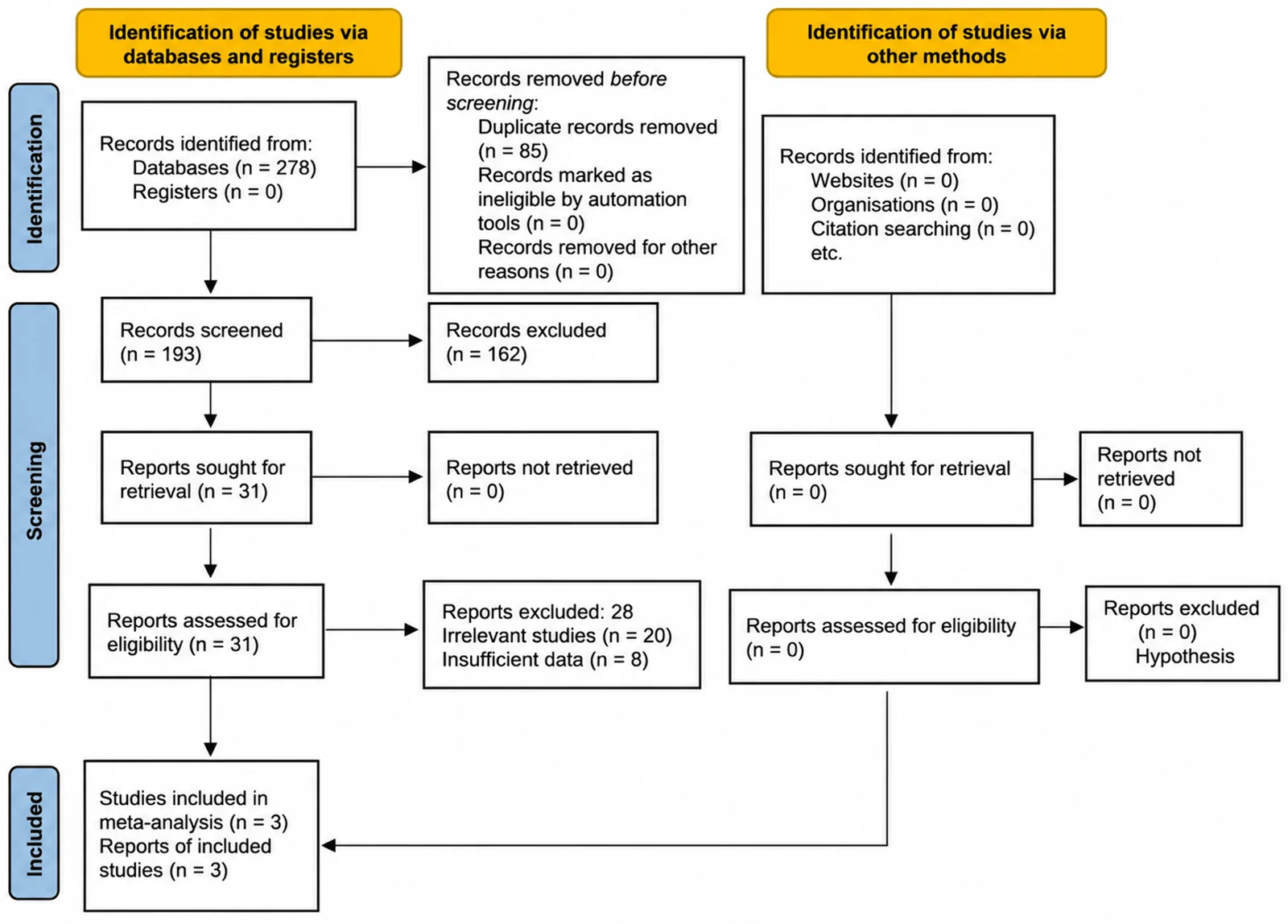
The PRISMA flow chart of the study selection process. PRISMA: Preferred Reporting Items for Systematic reviews and Meta-Analyses

A total of 278 articles were identified through database searches. After removing 85 duplicates, 193 articles remained for title and abstract screening. Of these, 162 articles were excluded as they did not meet the inclusion criteria, leaving 31 full-text articles for assessment. Following a detailed review, 28 articles were excluded due to irrelevance to the study criteria or insufficient data. Ultimately, three studies were included in the final analysis (Figure 1): the first by Schaefer et al. (2010) investigated a European population with aggressive periodontitis (AgP) [14], the second by Hashim et al. (2015) focused on a Middle Eastern population with AgP [16], and the third by Rodrigues et al. (2022) examined a Brazilian population with advanced periodontitis [15].

Characteristics of Included Studies

The studies represent diverse populations, genotyping methodologies, and disease subtypes, offering a comprehensive perspective on this genetic association.

In the study by Schaefer et al., German and Dutch populations with AgP were investigated [14]. This GWAS included 438 cases with generalized and localized AgP and 1,320 healthy controls. Cases were diagnosed based on severe clinical and radiographic criteria, characterized by attachment and bone loss typical of AgP. Controls were free of clinical attachment loss and served as a comparison group. High-throughput genotyping of rs1537415 was conducted as part of the GWAS, using the Affymetrix GeneChip (Thermo Fisher Scientific Inc., Waltham, Massachusetts, United States) and TaqMan (Thermo Fisher Scientific Inc.) SNP assays. The study examined the enrichment of the G allele in cases compared to controls, focusing on its statistical significance and ORs.

Hashim et al. investigated a Sudanese population with AgP [16]. A total of 132 cases and 136 healthy controls were included, and all non-smokers were matched for age and gender. Cases exhibited severe clinical attachment loss and alveolar bone destruction, while controls had no attachment loss or probing depths >3 mm. DNA was extracted from blood samples, and genotyping of rs1537415 was performed using the Sequenom MassARRAY iPLEX platform (CD Genomics, New York, United States). The analysis compared allele and genotype frequencies between cases and controls to evaluate the association of the G allele with AgP.

The study by Rodrigues et al. focused on a Brazilian population with advanced periodontitis [15]. A total of 100 cases were included, all classified as Stage III/IV based on the 2018 periodontal classification, with further stratification into Grade B (52 cases) and Grade C (48 cases). The control group comprised 61 healthy individuals with no clinical attachment loss or probing depths ≥3 mm. The controls were matched for age and gender to the cases. DNA was extracted from buccal epithelial cells, and genotyping of the rs1537415 polymorphism in the *GLT6D1* gene was performed using real-time quantitative PCR. Cases and controls were compared based on allele and genotype frequencies to assess the association of the G allele with advanced periodontitis.

These studies collectively covered distinct populations (Brazilian, German/Dutch, and Sudanese), representing both advanced chronic periodontitis (CP) and AgP. They utilized diverse genotyping platforms, including real-time PCR, GWAS, and MassARRAY, and employed rigorous diagnostic criteria for case and control definitions. Together, these methodologies and population characteristics provided a robust foundation for analyzing the association of the rs1537415 polymorphism with periodontitis (Tables 3, 4).

**Table 3:** General characteristics of the included studies. Classification adapted to 2018 AAP/EFP case definitions: “Aggressive periodontitis” reclassified as Stage III or IV, Grade C; “Chronic periodontitis” with severe involvement reclassified as Stage III or IV, Grades B or C. AAP: American Academy of Periodontology; EFP: European Federation of Periodontology; M: male; F: female; GWAS: genome-wide association studies

Study (Author, Year)	Country/Population	Sample Size (Cases / Controls)	Age, mean ± SD (years)	Gender (M/F)	Original Diagnostic Criteria	Current Classification (2018 AAP/EFP)*	Smoking Status	Systemic Conditions
Schaefer et al., 2010 [14]	German & Dutch	438 / 1320	NR in GWAS; cases ≤ 35 y	NR	Aggressive periodontitis (generalized/localized), severe attachment and bone loss (1999 AAP)	Stage III/IV, Grade C	NR	NR
Hashim et al., 2015 [16]	Sudanese	132 / 136	Cases: 27.9 ± 5.6; Controls: 27.8 ± 5.6	Cases: 71 M / 61 F; Controls: 76 M / 60 F	Aggressive periodontitis (1999 AAP)	Stage III/IV, Grade C	Non-smokers only	NR
Rodrigues et al., 2022 [15]	Brazilian	100 / 61	Cases: 50.2 ± 8.4; Controls: 49.3 ± 8.7	Cases: 37 M / 63 F; Controls: 20 M / 41 F	Stage III/IV, Grade B or C (2018 AAP/EFP)	Stage III/IV, Grade B or C	Non-smokers only	None included

**Table 4:** Outcomes of the included studies.

Study	SNP (Gene)	Outcome	G Allele Frequency	Originally reported OR (allelic)
Schaefer et al. [14]	rs1537415 (*GLT6D1*)	Significant association with aggressive periodontitis	Cases: 48.4% Controls: 38.8%	1.59 (95% CI: 1.36-1.86)
Hashim et al. [16]	rs1537415 (*GLT6D1*)	Significant association with aggressive periodontitis	Cases: 37.8% Controls: 28.8%	1.50 (95% CI: 1.04-2.17)
Rodrigues et al. [15]	rs1537415 (*GLT6D1*)	No significant association	Cases: 66.7% (Group B), 62.8% (Group C) Controls: 62.3%	Not significant

Risk of Bias

The Q-Genie assessment highlights the variability in the quality of the included studies (Table 5). Schaefer et al. achieved high scores across all domains, demonstrating robust methodology, adequate sample size, and substantial population stratification control, making it the most reliable study [14]. Hashim et al. showed moderate quality, with strengths in hypothesis framing but constrained by small sample size and inadequate control for population diversity [16]. Rodrigues et al. exhibited moderate-to-low quality, facing limitations in statistical power and stratification adjustment due to its small sample size [15]. These findings underscore the necessity for extensive, standardized studies to enhance reliability and generalizability.

**Table 5:** Q-Genie tool for risk of bias assessment.

Domain	Schaefer et al. [14]	Hashim et al. [16]	Rodrigues et al. [15]
Adequacy of Hypothesis and Rationale	High	High	Moderate
Classification and Measurement of Outcomes	High	Moderate	Moderate
Description and Comparison of Study Groups	High	Moderate	Moderate
Disclosure of Potential Sources of Bias	High	Moderate	Moderate
Statistical Methods and Power Analysis	High	Moderate	Low

Quantitative Synthesis

**Figure 2:**
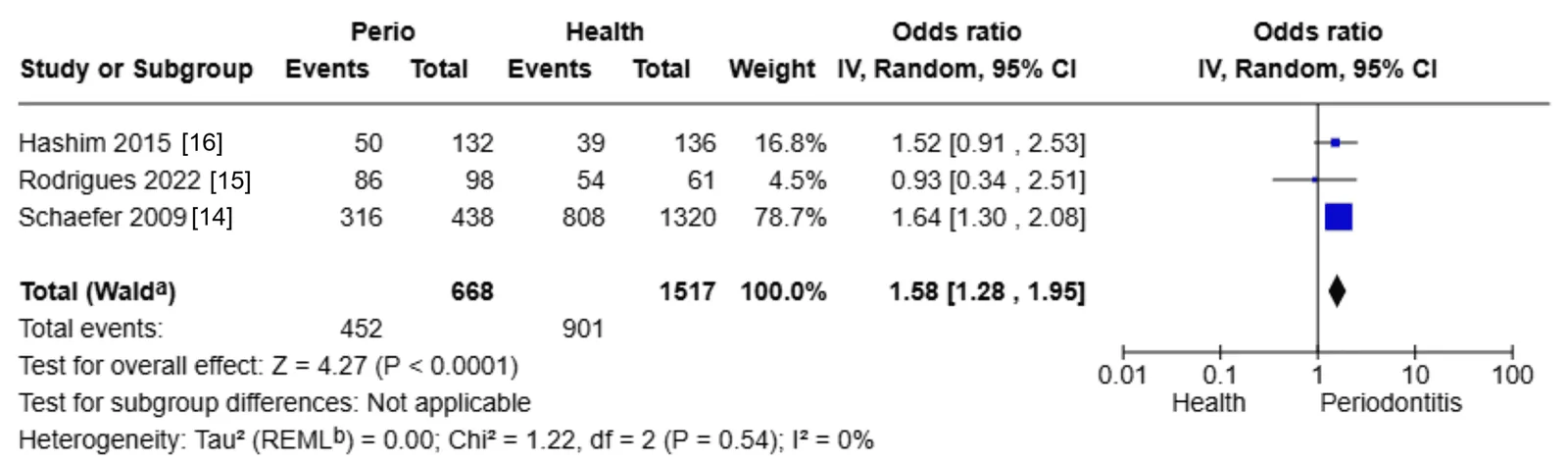
Forest plot displaying the meta-analysis of the association between the GLT6D1 rs1537415 G allele and periodontitis risk. ^a^CI calculated by Wald-type method; ^b^Tau^2^ calculated by Restricted Maximum-Likelihood Method

Data from the three included studies, comprising 2,185 participants (668 periodontitis cases and 1,517 healthy controls), were pooled to evaluate the association between the *GLT6D1* rs1537415 G allele and periodontitis risk. The random-effects meta-analysis demonstrated a statistically significant association, with an overall OR of 1.58 (95% CI: 1.28-1.95, p < 0.0001), indicating that carriers of the G allele have approximately 58% higher odds of developing Stage III/IV, Grade B or C periodontitis compared with non-carriers (Figure 2).

Study-specific results: Hashim et al. (2015): OR = 1.52, 95% CI: 0.91-2.53; association not statistically significant. Rodrigues et al. (2022): OR = 0.93, 95% CI: 0.34-2.51; no significant association observed. Schaefer et al. (2010): OR = 1.64, 95% CI: 1.30-2.08; statistically significant association. No statistically significant heterogeneity was detected (Tau² = 0.00; Chi² = 1.22, df = 2, p = 0.54; I² = 0%); however, this finding should be interpreted cautiously given the small number of included studies, as I² is imprecise and underpowered with only three studies.

Sensitivity Analysis

A sensitivity analysis excluding the Schaefer et al. (2010) GWAS (due to its large sample size and high statistical weight) [14] resulted in a pooled OR of 1.37 (95% CI: 0.87-2.16, p = 0.18), with no statistically significant association between rs1537415 and periodontitis. Heterogeneity remained low (I² = 0%). This indicates that the inclusion of Schaefer et al. [14] primarily drives the overall significant association observed in the primary analysis. With only three studies, assessment of publication bias was not reliable, and no firm conclusions could be drawn.

Discussion

Principal Findings

This meta-analysis demonstrated a significant association between the *GLT6D1* rs1537415 G allele and susceptibility to severe periodontitis, particularly Stage III/IV and Grade B or C, in European and Sudanese populations. The pooled OR of 1.58 (95% CI: 1.28-1.95, p < 0.0001) indicates that carriers of the G allele have approximately 58% higher odds of developing advanced forms of the disease compared to non-carriers. However, no statistically significant association was observed in the Brazilian cohort [15], suggesting possible population-specific differences in genetic architecture, allele frequencies, linkage disequilibrium patterns, or environmental exposures that may influence the effect of this variant on disease susceptibility. Sensitivity analysis excluding the large GWAS by Schaefer et al. [14] attenuated the association to a non-significant level (OR = 1.37, 95% CI: 0.87-2.16, p = 0.18), indicating that the overall effect is largely driven by this single study, which contributed nearly 79% of the total weight in the meta-analysis. This underscores the need for additional large-scale, well-powered studies to confirm the robustness of the observed association.

The association between rs1537415 and AgP was initially identified in European populations by Schaefer et al. [14], who reported an OR of 1.59 (95% CI: 1.36-1.86, p < 5.51 × 10⁻⁹) among German and Dutch individuals, and subsequently replicated in a Sudanese cohort by Hashim et al. [16], who found a similar OR of 1.50 (95% CI: 1.04-2.17). These results suggest a shared genetic predisposition across geographically and ethnically distinct groups. The lack of a significant association in the Brazilian sample may be explained by differences in environmental exposures, lifestyle factors such as diet or oral hygiene habits, and the complex admixture background of Brazilian populations, which could alter the linkage disequilibrium structure surrounding* GLT6D1* [15]. Moreover, phenotypic heterogeneity likely contributes to these disparities, as Brazilian cases were diagnosed under the 2018 AAP/EFP classification system [21] with a mix of Grade B and Grade C patients, whereas the European and Sudanese cohorts consisted solely of aggressive periodontitis cases diagnosed according to the 1999 AAP criteria [22].

Biological Plausibility

From a mechanistic perspective, the rs1537415 variant is located within the second intron of *GLT6D1* in a predicted binding site for the GATA-3 transcription factor. Functional studies suggest that the G allele reduces GATA-3 binding affinity [14], which is thought to impair T-helper 2 (Th2) cell differentiation and decrease production of anti-inflammatory cytokines such as IL-4, IL-5, and IL-13. This is proposed to favor a T-helper 1/T-helper 17 (Th1/Th17)-dominant immune profile, with elevated IL-1β, TNF-α, and matrix metalloproteinase activity [10]. Such an imbalance is thought to promote chronic inflammation, extracellular matrix degradation, and alveolar bone resorption, all hallmarks of severe periodontitis. *GLT6D1 *is involved in glycosylation processes considered critical for epithelial barrier integrity, immune receptor signaling, and extracellular matrix stability, and its reduced function could therefore plausibly compromise both host defense and tissue repair in periodontal tissues. The results of this meta-analysis align with previous GWASs that have implicated *GLT6D1 *and other immune-modulatory loci in the pathogenesis of periodontitis [10]. However, the magnitude and direction of association appear to vary across populations, indicating the possible influence of gene-environment interactions and the need for ethnicity-specific risk assessments. The consistent effect observed in European [14] and Sudanese [16] populations supports the hypothesis of a shared biological mechanism, whereas the null finding in the Brazilian cohort [15] underscores the complexity of genetic risk in admixed populations.

Strengths and Limitations

This review followed PRISMA guidelines, used a comprehensive multi-database search strategy, and applied independent dual-reviewer screening with a calibration exercise and third-reviewer adjudication, alongside a structured quality assessment (Q-Genie) of each included study, features that support the methodological rigor of the synthesis. Several limitations must nonetheless be considered when interpreting these findings. Only three studies met the inclusion criteria, which limits the statistical power of the pooled analyses and restricts the ability to perform extensive subgroup or meta-regression analyses to explore sources of heterogeneity. The effect estimate was heavily influenced by the largest included study [14], and exclusion of this study rendered the association non-significant, indicating that the observed effect is sensitive to the inclusion of this single dataset. There was limited representation of non-European populations, with only one study each from Sudan [16] and Brazil [15], reducing the generalizability of the results to other ethnic groups. Heterogeneity in diagnostic criteria was present across studies: the European and Sudanese cohorts were classified under the 1999 AAP aggressive periodontitis definition [22], while the Brazilian study used the 2018 AAP/EFP classification [21], which may have introduced phenotypic variability that could attenuate genetic associations. The genotyping platforms varied, ranging from high-density GWAS arrays to real-time PCR and MassARRAY, which, despite all meeting quality control standards, differ in sensitivity, allele calling algorithms, and potential for batch effects. The protocol for this meta-analysis was not prospectively registered in a database such as the International Prospective Register of Systematic Reviews (PROSPERO), which may limit transparency and introduce the potential for reporting bias [16]. Environmental and behavioral factors such as smoking, diet, systemic conditions, and oral microbiome composition were variably reported or unreported across studies, preventing adjustment for these important covariates and limiting the ability to assess gene-environment interactions [23,24]. Small sample sizes in the Sudanese [16] and Brazilian [15] studies increase the risk of type II error and limit the precision of their effect estimates.

In summary, this meta-analysis supports the association of the *GLT6D1* rs1537415 G allele with severe periodontitis in European and Sudanese populations [14,16], while highlighting a lack of association in Brazilian patients with advanced chronic periodontitis [15]. The proposed functional relevance of this allele, through its potential impact on GATA-3-mediated immune regulation, offers a biologically plausible, though not yet confirmed, mechanism for its role in disease susceptibility [14,25]. However, given the small number of available studies, the predominance of European ancestry data, variability in phenotype definitions, and methodological differences, the findings should be interpreted with caution. Future research should prioritize large, multi-ethnic cohorts with standardized case definitions and genotyping methods, incorporate detailed environmental exposure data, and conduct functional studies to clarify the role of *GLT6D1* in immune modulation and periodontal disease progression [26,27].

## Conclusions

This meta-analysis suggests a possible association between the *GLT6D1* rs1537415 G allele and severe periodontitis. However, the evidence remains limited by the small number of studies and by the loss of statistical significance when the largest study is excluded in a sensitivity analysis. Further large, multi-ethnic studies with standardised case definitions are needed to confirm this association before it can be regarded as a validated risk marker. In practice, early identification of individuals carrying high-risk alleles such as rs1537415 could be integrated with established clinical and behavioural risk assessments to refine risk stratification. This might involve targeted genetic screening for patients with a family history of severe periodontitis or in populations with higher allele frequencies. Identified high-risk individuals could receive more intensive preventive interventions (e.g., shorter recall intervals, tailored oral hygiene instructions, adjunctive antimicrobial therapies) and earlier initiation of treatment when signs of disease appear. This approach would differ from the current standard of care, which primarily initiates more intensive management only after clinical disease presentation rather than in the pre-clinical phase. However, implementation would require robust validation of the genetic marker across multiple ethnicities, cost-effectiveness analyses, and integration into existing periodontal risk assessment frameworks before routine clinical adoption.

Future studies should focus on expanding multi-ethnic cohorts to validate the association of the *GLT6D1 *rs1537415 G allele with periodontitis across diverse populations. Standardizing diagnostic criteria and genotyping methodologies will be critical to reducing inter-study variability. Additionally, further research should investigate the gene-environment interactions that may modulate this allele's effects, including factors such as smoking, systemic diseases, and oral microbiome composition. Functional studies exploring the biological mechanisms underlying *GLT6D1*-mediated immune dysregulation and inflammation could also provide valuable insights for developing targeted therapeutic interventions.

## Appendices

**Table 6:** PRISMA checklist. PRISMA: Preferred Reporting Items for Systematic Reviews and Meta-Analyses

Section and Topic	Item #	Checklist Item
TITLE	1	Identify the report as a systematic review.
ABSTRACT	2	See the PRISMA 2020 for Abstracts checklist.
INTRODUCTION	3	Describe the rationale for the review in the context of existing knowledge.
	4	Provide an explicit statement of the objective(s) or question(s) the review addresses.
METHODS	5	Specify the inclusion and exclusion criteria for the review and how studies were grouped for the syntheses.
	6	Specify all databases, registers, websites, organisations, reference lists, and other sources searched or consulted to identify studies. Specify the date when each source was last searched or consulted.
	7	Present the full search strategies for all databases, registers, and websites, including any filters and limits used.
	8	Specify the methods used to decide whether a study met the inclusion criteria of the review, including how many reviewers screened each record and each report retrieved, whether they worked independently, and if applicable, details of automation tools used in the process.
	9	Specify the methods used to collect data from reports, including how many reviewers collected data from each report, whether they worked independently, any processes for obtaining or confirming data from study investigators, and if applicable, details of automation tools used in the process.
	10a	List and define all outcomes for which data were sought. Specify whether all results that were compatible with each outcome domain in each study were sought (e.g. for all measures, time points, analyses), and if not, the methods used to decide which results to collect.
	10b	List and define all other variables for which data were sought (e.g. participant and intervention characteristics, funding sources). Describe any assumptions made about any missing or unclear information.
	11	Specify the methods used to assess risk of bias in the included studies, including details of the tool(s) used, how many reviewers assessed each study and whether they worked independently, and if applicable, details of automation tools used in the process.
	12	Specify for each outcome the effect measure(s) (e.g. risk ratio, mean difference) used in the synthesis or presentation of results.
	13a	Describe the processes used to decide which studies were eligible for each synthesis.
	13b	Describe any methods required to prepare the data for presentation or synthesis, such as handling of missing summary statistics, or data conversions.
	13c	Describe any methods used to tabulate or visually display results of individual studies and syntheses.
	13d	Describe any methods used to synthesize results and provide a rationale for the choice(s). If meta-analysis was performed, describe the model(s), method(s) to identify the presence and extent of statistical heterogeneity, and software package(s) used.
	13e	Describe any methods used to explore possible causes of heterogeneity among study results.
	13f	Describe any sensitivity analyses conducted to assess robustness of the synthesized results.
	14	Describe any methods used to assess risk of bias due to missing results in a synthesis (arising from reporting biases).
	15	Describe any methods used to assess certainty (or confidence) in the body of evidence for an outcome.
RESULTS	16a	Describe the results of the search and selection process, from the number of records identified in the search to the number of studies included in the review, ideally using a flow diagram.
	16b	Cite studies that might appear to meet the inclusion criteria, but which were excluded, and explain why they were excluded.
	17	Cite each included study and present its characteristics.
	18	Present assessments of risk of bias for each included study.
	19	For all outcomes, present, for each study: summary statistics for each group and an effect estimate and its precision, ideally using structured tables or plots.
	20a	For each synthesis, briefly summarise the characteristics and risk of bias among contributing studies.
	20b	Present results of all statistical syntheses conducted, including summary estimates and measures of statistical heterogeneity.
	20c	Present results of all investigations of possible causes of heterogeneity among study results.
	20d	Present results of all sensitivity analyses conducted.
	21	Present assessments of risk of bias due to missing results (arising from reporting biases).
	22	Present assessments of certainty (or confidence) in the body of evidence for each outcome assessed.
DISCUSSION	23a	Provide a general interpretation of the results in the context of other evidence.
	23b	Discuss any limitations of the evidence included in the review.
	23c	Discuss any limitations of the review processes used.
	23d	Discuss implications of the results for practice, policy, and future research.
OTHER INFORMATION	24a	Provide registration information for the review, or state that the review was not registered.
	24b	Indicate where the review protocol can be accessed, or state that a protocol was not prepared.
	24c	Describe and explain any amendments to information provided at registration or in the protocol.
	25	Describe sources of financial or non-financial support for the review and the role of the funders.
	26	Declare any competing interests of review authors.
	27	Report which data, code, or other materials are publicly available and where they can be found.
